# Acupuncture for headache in COVID-19: A protocol for systematic review and meta-analysis

**DOI:** 10.1097/MD.0000000000028174

**Published:** 2021-12-10

**Authors:** Mi Sun, Xian Jin, Mingxiao Zang, Weijia Jiang, Chunxiao Zhao, Jieyu Bi, Huijuan Yu, Qiwen Tan

**Affiliations:** a Department of Acupuncture and Moxibustion, Shandong University of Traditional Chinese Medicine, Jinan, Shandong, China; b Affiliated Hospital of Shandong University of Traditional Chinese Medicine, Jinan, Shandong, China; c Shandong University of Traditional Chinese Medicine, Jinan, Shandong, China.

**Keywords:** acupuncture, coronavirus disease 2019, headache, protocol

## Abstract

**Background:**

Coronavirus disease 2019 (COVID-19) is an acute respiratory infectious disease which making people difficult to breathe and often accompanied with headache. Acupuncture have been proved the therapeutic effect on headache, but there has been no high-quality evidence on acupuncture for the headache in COVID-19. This study is designed to evaluate the effectiveness and safety of acupuncture for headache in COVID-19.

**Methods:**

Randomized controlled trials from December 2019 to July 2021 will be included without restrictions on language or publication date. PubMed, EMBASE, Cochrane Library, Web of Science, Chinese Biomedical Databases, China National Knowledge Infrastructure, Wanfang database, and VIP database will be searched. Two researchers will independently select studies, extract data and evaluate study quality. Cochrane risk of bias tool for randomized trials will be used to assess the risk of bias of included studies. Statistical analyses will be performed using the Review Manager V.5.3 and stata 14.0.

**Ethics and dissemination:**

This study will not involve personal information. Ethical approval will not be required. We will publish the results in a peer-reviewed journal.

**PROSPERO Trial registration number:**

CRD42021270722

## 1 Introduction

Coronavirus disease 2019 (COVID-19) is caused by severe acute respiratory syndrome coronavirus 2 (SARS-CoV-2) infection with high infectivity and tall mortality, and it has rapidly developed into a worldwide public health emergencies in the world. The novel coronavirus pneumonia patients not only show respiratory symptoms such as dyspnea, but also have nervous system symptoms such as headache and dizziness.^[1,2]^ Research has indicated that headache is one of the most common neurologic symptoms, with a prevalence of about 10%.^[3–5]^ The National Institutes of Health noted that headache is one of the common symptoms of postacute sequelae of SARS-CoV-2 infection.^[6]^ Meanwhile, according to the latest report, headache is the most common symptoms for delta variant cases. Headache may be worsen or start after major stressful life events or posttraumatic stress disorder. Nowadays, the treatment methods for headache of COVID-19 are mainly western medicines which may prolong viral replication in SARS-CoV2 and might be associated with a worse COVID-19 clinical course.^[7]^ Therefore, alleviate the headache in COVID-19 patients is helpful to improve the quality of life.

Acupuncture is one of the external treatments in Traditional Chinese Medicine with a history of more than 3000 years. Studies have proved that acupuncture has unique advantages in the treatment of headache and was widely used in worldwide.^[8–10]^ During the COVID-19 epidemic, acupuncture has been used as a complementary treatment for COVID-19 in China and has been confirmed the efficacy of COVID-19 with routine regimens.^[11]^

It has been demonstrated that dopamine along with its D1 and D2 receptors participates in electroacupuncture-induced analgesia and lung immunity might be shaped by dopamine receptors.^[12]^ Differing from the opioids and other medicines, which may exacerbate the symptoms of gastrointestinal disorders, acupuncture may be a safe and effective method for pain management in COVID-19 patients.^[12,13]^

As of yet, there has been no high-quality evidence on acupuncture for the headache in COVID-19. Therefore, we designed this study to better understanding of the effectiveness and safety of acupuncture therapy for headache in COVID-19.

## 2 Methods

### 2.1 Study registration

This systematic review protocol has been registered in the PROSPERO (No. CRD42021270722). We will follow recommendations outlined in The Cochrane Handbook of Systematic Review of Interventions and the preferred reporting items for systematic reviews and meta-analysis protocol (PRISMA-P) statement guidelines. If amendments are needed, we will update our protocol to include any changes in the whole process of research.

### 2.2 Types of studies

Randomized controlled trails will be included, without restrictions on language or publication date.

### 2.3 Types of participants

Subjects with documented COVID-19 with headache of 4 weeks duration or longer. There is no restrictions on gender, race, and disease stage. Patients with history of headache prior to COVID-19 infection will be excluded.

### 2.4 Types of interventions and comparisons

In addition to the treatment of COVID-19, treatment group interventions comprised acupuncture, electro-acupuncture, auricular acupuncture, or laser acupuncture, and comparator groups intervention: comfort therapy (placebo, pseudo-acupuncture or blank control), other therapies (Western medicine, usual care or nondrug therapy, etc).

### 2.5 Types of outcomes

Outcomes include effectiveness indicators and safety indicators.

#### 2.5.1 Effectiveness indicators

Clinical variables will be set as the effectiveness indicators, such as the headache frequency, headache intensity, duration of headache, times of using painkiller, and quality of life.

#### 2.5.2 Safety indicator

The incidence of adverse events.

### 2.6 Search methods for identification of studies

Randomized controlled trials will be extracted from PubMed, EMBASE, Cochrane Library, Web of Science, Chinese Biomedical Databases, China National Knowledge Infrastructure, Wanfang database, and VIP database. The complete PubMed search strategy is summarized in Table 1.

**Table 1:** PubMed search strategy.

Number	Search items
#1	“covid 19”[Title/Abstract] OR “2019-nCoV”[Title/Abstract] OR “coronavirus disease 19”[Title/Abstract] OR “2019 novel coronavirus”[Title/Abstract] OR “coronavirus disease 2019”[Title/Abstract] OR “disease 2019 coronavirus”[Title/Abstract] OR “sars coronavirus 2 infection”[Title/Abstract] OR “SARS-CoV-2”[Title/Abstract]
#2	“acupuncture”[Title/Abstract] OR “moxibustion”[Title/Abstract] OR “electroacupuncture”[Title/Abstract] OR “fire needle”[Title/Abstract] OR “acupoint injection”[Title/Abstract] OR “auricular point”[Title/Abstract] OR “warming needle moxibustion”[Title/Abstract]
#3	“Headaches”[Title/Abstract] OR “Head”[Title/Abstract] OR “Cephalodynia”[Title/Abstract] OR “Cranial”[Title/Abstract] OR “Cephalalgia”[Title/Abstract] OR “Cephalgia”[Title/Abstract]
#4	#1 and #2 and #3

### 2.7 Data collection

#### 2.7.1 Selection of studies

**Figure 1:**
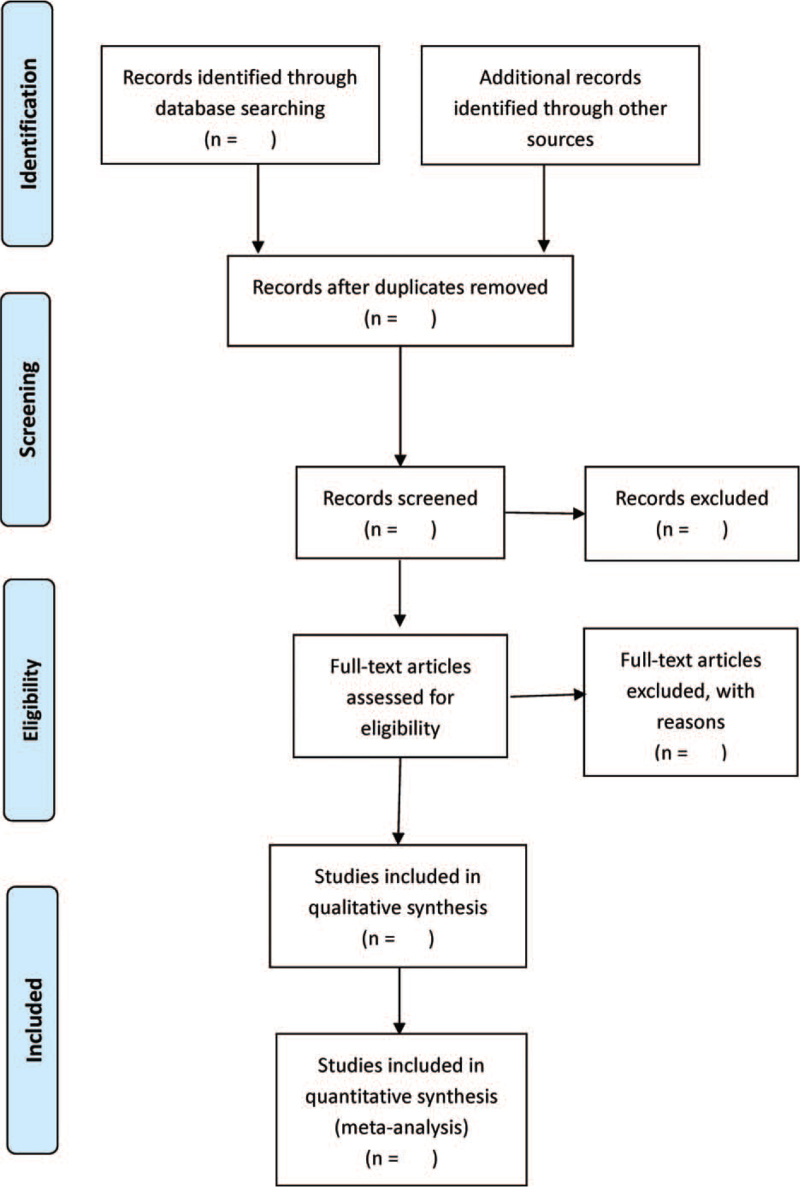
PRISMA flow diagram. PRISMA = preferred reporting items for systematic reviews and meta-analysis.

Two reviewers (MS and JX) will search the study independently, and then they will screen the studies by reviewing titles and abstracts or full text if necessary. Further unresolved discrepancy was managed by a third reviewer (MXZ). The selection process is summarized using PRISMA flow diagram. Details of the selection procedure for studies are shown in a PRISMA flow chart (Fig. 1).

#### 2.7.2 Data extraction and management

Data will then be extracted from the selected studies by 2 reviewers (WJJ and CXZ). The detailed extraction information are as follows: basic information of the included studies, baseline characteristics of the subjects, intervention measures and control measures, key elements of bias risk assessment, outcome indicators, etc. Any disagreements will be resolved by discussion, or by consultation with a third author (XMZ).

Endnote X9.3 will be used to manage the search results and perform screening and the duplicate publications will be excluded.

#### 2.7.3 Quality and bias assessment

The methodological quality and risk of bias of each trial will be assessed by 2 review authors (JYB and CXZ) independently according to the Cochrane Handbook. The following characteristics will be assessed: random sequence generation, allocation concealment, blinding of participants and personnel, blinding of outcome assessment, incomplete outcome data, selective reporting, and other bias. Based on the assessments of the studies against these 7 domains, they will be classified as being of “low risk”, “high risk”, or “unclear risk” of bias. Any disagreements will be resolved by discussion, or by consultation with another reviewer (XMZ).

#### 2.7.4 Dealing with missing data

If complete literature or relevant data is not available, we will contact the corresponding author. However, if the missing data cannot be obtained, then the study will be excluded from the analysis.

### 2.8 Statistical analysis

#### 2.8.1 Measures of treatment effect

Review Manager (RevMan 5.3) software will be used to conduct this meta-analysis.

Dichotomous outcomes will be presented as risk ratios with 95% confidence intervals. When continuous outcomes exists, mean differences or standardized mean differences will be calculated.

#### 2.8.2 Assessment of heterogeneity

The choice of whether to conduct a meta-analysis and which model to use (fixed or random effects) will depend on the level of statistical heterogeneity assessed by the *P* value and *I*^*2*^ index. *P *< .05 considered to represent significant statistical heterogeneity, and *I*^*2*^ >50% considered to be indicative of substantial heterogeneity.

#### 2.8.3 Data synthesis

The fixed effect model will be used if no significant heterogeneity is observed; otherwise, the random effect model will be applied for statistical analysis.^[14]^

#### 2.8.4 Subgroup analysis

According to the results of the data synthesis, we will perform subgroup analyses, or a meta-regression to analyze the source of any heterogeneity.

#### 2.8.5 Assessment of reporting bias

When outcomes include more than 10 studies, we will use Stata 14.0 to access the reporting bias by funnel plot and Egger test.^[15]^

#### 2.8.6 Sensitivity analysis

Sensitivity analyses will be performed to determine whether the results are affected by leave1out with Stata14.0.

#### 2.8.7 Quality of evidence evaluation

The evidence quality will be evaluated by 2 viewers (MS and JX) independently with the grading of recommendations assessment, development and evaluation. According to 5 parameters (publication bias, indirectness, inconsistency, imprecision, and study limitations), evidence quality will be rated “high”, “moderate’,’ “low” according to the rating standards.

#### 2.8.8 Ethics and dissemination

Since this study does not involve the patient privacy, ethical approval is not required. Our research results will be shared and shown through conference reports and peer-reviewed journals.

## 3 Discussion

COVID-19 caused by SARS-CoV-2, which has become a serious public health threat worldwide, with millions of people at risk in increasing countries.^[16]^ Dyspnea, fatigue, fever and cough were highly prevalent in COVID-19, however, the COVID-19 pandemic has brought numerous patients complaining of neurologic manifestations, such as headache and dizziness.^[17–19]^ Headache can lead to worsening symptoms, and intensification of negative emotions.^[20]^

Acupuncture was regarded as a complementary technique, and was widely applied in treating headache. Acupuncture is convenient, simple, and low in cost. It is benefit for COVID-19 patients with headache to accept acupuncture treatment. Thus, this study aimed to provide evidence of acupuncture therapy for headache in COVID-19 and aided treatment decisions.

## Author contributions

**Conceptualization:** Mi Sun.

**Formal analysis:** Xian Jin.

**Investigation:** Mingxiao Zang.

**Resources:** Chunxiao Zhao.

**Software:** Weijia Jiang.

**Visualization:** Huijuan Yu.

**Writing – original draft:** Mi Sun, Jieyu Bi.

**Writing – review & editing:** Qiwen Tan.

## References

[1] Collantes M Espiritu AI Sy M Anlacan V Jamora R. Neurological Manifestations in COVID-19 Infection: a systematic review and meta-analysis. Can J Neurol Sci 2021;48:66–76. 32665054 10.1017/cjn.2020.146 PMC7492583

[2] Tsai ST Lu MK San S Tsai CH. The neurologic manifestations of coronavirus disease 2019 pandemic: a systemic review. Front Neurol 2020;11:498. 32574246 10.3389/fneur.2020.00498 PMC7248254

[3] Roy D Ghosh R Dubey S Dubey MJ Benito-Leon J Kanti RB. Neurological and neuropsychiatric impacts of COVID-19 pandemic. Can J Neurol Sci 2021;48:09–24. 10.1017/cjn.2020.173 PMC7533477 32753076

[4] Mao L Jin H Wang M. Neurologic manifestations of hospitalized patients with coronavirus disease 2019 in Wuhan, China. JAMA Neurol 2020;77:683–90. 32275288 10.1001/jamaneurol.2020.1127 PMC7149362

[5] Chen N Zhou M Dong X. Epidemiological and clinical characteristics of 99 cases of 2019 novel coronavirus pneumonia in Wuhan, China: a descriptive study. Lancet 2020;395:507–13. 32007143 10.1016/S0140-6736(20)30211-7 PMC7135076

[6] Moghimi N Di Napoli M Biller J. The neurological manifestations of post-acute sequelae of SARS-CoV-2 infection. Curr Neurol Neurosci Rep 2021;21:44. 34181102 10.1007/s11910-021-01130-1 PMC8237541

[7] Bobker SM Robbins MS. COVID-19 and headache: a primer for trainees. Headache 2020;60:1806–11. 32521039 10.1111/head.13884 PMC7300928

[8] Zhang R Lao L Ren K Berman BM. Mechanisms of acupuncture-electroacupuncture on persistent pain. Anesthesiology 2014;120:482–503. 24322588 10.1097/ALN.0000000000000101 PMC3947586

[9] Vickers AJ Vertosick EA Lewith G. Acupuncture for chronic pain: update of an individual patient data meta-analysis. J Pain 2018;19:455–74. 29198932 10.1016/j.jpain.2017.11.005 PMC5927830

[10] Millstine D Chen CY Bauer B. Complementary and integrative medicine in the management of headache. BMJ 2017;357:j1805. 28512119 10.1136/bmj.j1805

[11] Badakhsh M Dastras M Sarchahi Z Doostkami M Mir A Bouya S. Complementary and alternative medicine therapies and COVID-19: a systematic review. Rev Environ Health 2021;36:443–50. 33838089 10.1515/reveh-2021-0012

[12] Han Z Zhang Y Wang P Tang Q Zhang K. Is acupuncture effective in the treatment of COVID-19 related symptoms? Based on bioinformatics/network topology strategy. Brief Bioinform 2021;doi: 10.1093/bib/bbab110. 10.1093/bib/bbab110 PMC8083275 33866350

[13] Drozdzal S Rosik J Lechowicz K. COVID-19: pain management in patients with SARS-CoV-2 infection-molecular mechanisms, challenges, and perspectives. Brain Sci 2020;10:07. 10.3390/brainsci10070465 PMC7407489 32698378

[14] Yang M Hu Z Yue R Yang L Zhang B Chen Y. The efficacy and safety of qiming granule for dry eye disease: a systematic review and meta-analysis. Front Pharmacol 2020;11:580. 32425798 10.3389/fphar.2020.00580 PMC7204435

[15] Egger M Davey SG Schneider M Minder C. Bias in meta-analysis detected by a simple, graphical test. BMJ 1997;315:629–34. 9310563 10.1136/bmj.315.7109.629 PMC2127453

[16] Majumder J Minko T. Recent developments on therapeutic and diagnostic approaches for COVID-19. AAPS J 2021;23:14. 33400058 10.1208/s12248-020-00532-2 PMC7784226

[17] Yachou Y El IA Belapasov V Ait BS. Neuroinvasion, neurotropic, and neuroinflammatory events of SARS-CoV-2: understanding the neurological manifestations in COVID-19 patients. Neurol Sci 2020;41:2657–69. 32725449 10.1007/s10072-020-04575-3 PMC7385206

[18] Bolay H Gul A Baykan B. COVID-19 is a real headache!. Headache 2020;60:1415–21. 32412101 10.1111/head.13856 PMC7272895

[19] Kim GU Kim MJ Ra SH. Clinical characteristics of asymptomatic and symptomatic patients with mild COVID-19. Clin Microbiol Infect 2020;26:941–8. 10.1016/j.cmi.2020.04.040 PMC7252018 32360780

[20] Rizzoli P Mullally WJ. Headache. Am J Med 2018;131:17–24. 28939471 10.1016/j.amjmed.2017.09.005

